# Supplementary material for: The genome sequence of an ichneumonid wasp, Netelia melanura (Thomson, 1888) (Hymenoptera: Ichneumonidae)
Source: Wellcome Open Res. 2025 Oct 6;10:556. [Version 1] doi: 10.12688/wellcomeopenres.24927.1 (PMC12569507; doi:10.12688/wellcomeopenres.24927.1)
Supplement: Peer review history

# Reviewer report: Reviewer response for version 1

**DOI:** 10.21956/wellcomeopenres.27457.r135385

**Published:** 2025-11-20

**Authors:** Bhagya C. Thimmappa

**Affiliations:** 1 USDA-ARS Beltsville Agricultural Research Center, Beltsville, Maryland, USA

In this article, the authors perform whole-genome sequencing of the male ichneumonid wasp, *Netelia melanura,* and report the first nuclear and mitochondrial genome assemblies. The reported nuclear genome assembly at the chromosome level is 253.87 megabases in size, and the mitochondrial genome assembly is 28.04 kilobases in length. High-quality assembly was achieved using PacBio HiFi and Hi-C reads, followed by manual curation, resulting in a 94.9% BUSCO value. The data generated in this study have been made publicly available.

 Minor comments:

1. In Table 2, please provide a hyperlink to the assembly accession number for easy access.
2. Correct the Figure 4 caption.
3. I see that transcriptome data are generated and made publicly available. However, providing nuclear and mitochondrial genome annotation information would further facilitate molecular and evolutionary studies.

Are sufficient details of methods and materials provided to allow replication by others?

Yes

Is the rationale for creating the dataset(s) clearly described?

Yes

Are the datasets clearly presented in a useable and accessible format?

Yes

Are the protocols appropriate and is the work technically sound?

Yes

Reviewer Expertise:

Bioinformatics, genomics, transcriptomics, multi-omics

I confirm that I have read this submission and believe that I have an appropriate level of expertise to confirm that it is of an acceptable scientific standard.

# Reviewer report: Reviewer response for version 1

**DOI:** 10.21956/wellcomeopenres.27457.r135788

**Published:** 2025-10-28

**Authors:** Alejandro Zaldívar Riverón

**Affiliations:** 1 Universidad Nacional Autónoma de México, Ciudad de Mexico, Mexico

In this article the authors report a complete nuclear genome sequence of the ichneumonid wasp species *Netelia melanura*. This genome assembly was conducted  as in previous published studies carried out by the first author. The genome sequence report is comprehensive and the mitochondrial genome was also assembled. I recommend this manuscript to be accepted for its indexing in Welcome Open Research

Are sufficient details of methods and materials provided to allow replication by others?

Yes

Is the rationale for creating the dataset(s) clearly described?

Yes

Are the datasets clearly presented in a useable and accessible format?

Yes

Are the protocols appropriate and is the work technically sound?

Yes

Reviewer Expertise:

Systematics, molecular evolution

I confirm that I have read this submission and believe that I have an appropriate level of expertise to confirm that it is of an acceptable scientific standard.

# Reviewer report: Reviewer response for version 1

**DOI:** 10.21956/wellcomeopenres.27457.r135382

**Published:** 2025-10-21

**Authors:** Alexandra Cerqueira De Araujo

**Affiliations:** 1 University College London, London, UK

The authors present here a chromosome-level assembly of the genome of *Netelia melanura *(Ichneumonidae, Hymenoptera), obtained from a single haploid male. This is the first assembly available for this species (three other chromosome-level assemblies are available on NCBI for this genus). This assembly is composed of 7 chromosome-level pseudomolecules (comprising 94.81% of the total assembly) and one mitochondrial chromosome. The assembly has a total length of 253.87 Mb and a scaffold N50 of 39.45 Mb. This assembly achieves an EBP reference standard of 5.C.Q58 and contains most of the orthologous genes found in hymenopterans (i.e. 94.9% of the complete BUSCO sequences). The code used to produce the data is available here https://github.com/sanger-tol.

 Comments:

1. The authors mentioned that a close relative of *N. melanura* has a karyotype of 6 chromosomes. On NCBI, 3 other *Netelia* species have a genome assembly with 6 chromosomal pseudomolecules ( *N. virgata*, *N. fuscicronis* and *N. thomsonii*, all resulting from the DToL project). In Gokhman V.E., 2011 (DOI: 10.15298/rusentj.20.3.09), Gokhman mentioned that no karyotype of 6 chromosomes was available at the time in parasitoid wasps. Then, Gokhman  mentioned that the karyotype of *Netelia latungula* shows 7 chromosomes. Is there any reference for this close relative of *N. melanura* showing its karyotype of 6 chromosomes?
2. In the “Genome sequence report”: “Sequence data” section, it says that the repeat content of the haploid genome is 16.13% (followed by a link to Figure 2). I don’t see where this result can be obtained on figure 2. Should it not be around 15.6%? For demonstration, 84.4% of the assembly is composed of unique content, 100-84.4 = 15.6.
3. In the “Genome sequence report”: “Sequence data” section, the authors say that “the sequencing data provided approximately 83x coverage”. Should it not be 93x (20.40e+9/218.52e+6)? Unless some reads have been filtered before the analysis or another way of calculating the coverage has been used. If the total span of the assembly has been used for the calculation, the sentence in the first paragraph of “Sequence data” shouldn’t say “Based on the estimated genome size, the sequencing data provided approximately 83× coverage.”, which comes just after the k-mer based estimation.
4. In legend of figure 4, it mentions green and blue curves that are not visible on the figure (as it should be since hymenopteran males are haploid). This should be removed from the legend. 
5. In the “Genome sequence report” section, the main text (I’m excluding the tables and figures here) contains software names with sometimes the version (e.g. BUSCO) and sometimes without it (e.g. Merqury.FK). This should be standardised throughout the text of this section.
6. Observation about the Hi-C map: if, within a chromosome, the contactless portions represent centromeres, it seems that the chromosomes arms around the centromere of the same chromosome don’t spatially interact with each other. There are some levels of contact in trans. It also looks like some portions could have been mis-joined and could be split or inverted.

Are sufficient details of methods and materials provided to allow replication by others?

Yes

Is the rationale for creating the dataset(s) clearly described?

Yes

Are the datasets clearly presented in a useable and accessible format?

Yes

Are the protocols appropriate and is the work technically sound?

Yes

Reviewer Expertise:

Comparative genomics

I confirm that I have read this submission and believe that I have an appropriate level of expertise to confirm that it is of an acceptable scientific standard.
